# Hamming Distance Method with Subjective and Objective Weights for Personnel Selection

**DOI:** 10.1155/2014/865495

**Published:** 2014-03-17

**Authors:** R. Md Saad, M. Z. Ahmad, M. S. Abu, M. S. Jusoh

**Affiliations:** ^1^Institute of Engineering Mathematics, Universiti Malaysia Perlis, Pauh Putra Main Campus, 02600 Arau, Perlis, Malaysia; ^2^School of Business Innovation and Technopreneurship, Universiti Malaysia Perlis, Jalan Kangar-Alor Setar, 01000 Kangar, Perlis, Malaysia

## Abstract

Multicriteria decision making (MCDM) is one of the methods that popularly has been used in solving personnel selection problem. Alternatives, criteria, and weights are some of the fundamental aspects in MCDM that need to be defined clearly in order to achieve a good result. Apart from these aspects, fuzzy data has to take into consideration that it may arise from unobtainable and incomplete information. In this paper, we propose a new approach for personnel selection problem. The proposed approach is based on Hamming distance method with subjective and objective weights (HDMSOW's). In case of vagueness situation, fuzzy set theory is then incorporated onto the HDMSOW's. To determine the objective weight for each attribute, the fuzzy Shannon's entropy is considered. While for the subjective weight, it is aggregated into a comparable scale. A numerical example is presented to illustrate the HDMSOW's.

## 1. Introduction

The rapid growth in globalization had created an intense competition between modern firms in global markets. These situations had urged the organization and firms to establish a comprehensive procedure during personnel selection process. The personnel selection can be defined as a process of selecting the individuals who match the requirement and qualification to perform a particular job in an excellent way [1]. The main objective of this process is to assess the diversity among the alternatives that could pave a way of predicting the future performance [2]. Knowing the fact that personnel selection is not an easy task to be solved has awakened the conscience of decision makers to make decisive action to solve. The decision makers have to consider all aspects that are needed in this process. Hence, some of the decision makers try to solve this problem by using any kind of methods that are available and suitable for them to use.

Despite restructuring and reorganizing the personnel selection process, some of the firms had performed a so-called “strategic decision” to choose the best candidate during the selection process. Some decision makers try to utilize rigorous and costly selection procedure and some even used the traditional method which depends on only information stated on the application forms that turn out to be quickest and inexpensive methods [1]. However, these methods actually never bring satisfaction and their final results are sometimes deniable. Thus, when multicriteria decision making (MCDM) was introduced in the early 1970's, it had become one of favorable and important methods in this area. Some of the decision makers took a chance and grabed this opportunity to apply this method in solving personnel selection problem [3]. MCDM is known for its capabilities in evaluating, electing, or ranking a finite set of available alternatives with respect to multiple and conflicting criteria [4]. A number of methods and theories had been introduced and extended based on the utilization of this approach and the continuing study of this field had extended in a fixed rate. Preference Ranking Organization Method for Enrichment Evaluation (PROMETHEE) [5], linear programming techniques [6], Analytic Hierarchy Process (AHP) [7], Simple Additive Weighting (SAW) [8], and Technique for Order Preference by Similarity to Ideal Solution or TOPSIS [9] are some of the numerous examples on MCDM methods that particularly have been used by the decision makers.

Distance measure can be identified as one of the MCDM approaches that can be used in personnel selection process. This approach holds an important key to solve many problems related to biology, science, social, and technology due to its capability of constructing some related distance measures, notably similarity, and proximity which always become a norm in various problems [10]. In recent years, the study of this method has been rapidly growing in which it resulted in proposing and improving the previous distance measure methods. Some of the well-known distance measure methods are Hamming, Euclidean, Hausdorff, and Minkowski methods. Based on the existing literatures, Hamming distance is one of the methods that can be used in personnel selection process [11–13]. This method was proposed by Hamming [14] in 1950 to count the number of flipping bits in a fixed-length binary word as an estimate of error used in telecommunication. Hamming distance is known for its ability in calculating the difference between two sets or elements. For example, the distance between interval-valued fuzzy sets. Consequently, apart from the decision making problem, it also has been applied in various fields such as communication [15], iris recognition [16], and engineering [17].

Literally, evaluation of certain criteria or attributes to select an appropriate alternative for specified position could become tremendous and challenging task for the decision makers. It is because some of the criteria such as leadership, personality, and creativity are referred to as qualitative criteria in which exhibits imprecise and vagueness data. In general, this uncertainty and subjective scene that occurs during the evaluation of the alternatives based on respective criteria and criteria weight may come from various sources including unquantifiable information, incomplete information, unobtainable information, and partial ignorance [18]. For this situation, commonly classical MCDM will be put aside since the alternatives rating and criteria weights for classical MCDM are usually measured in crisp numbers. Therefore, one of the best resorts to solve this problem is by applying fuzzy set theory. The fuzzy set theory is known for its flexibility in handling imprecise and uncertainty in human judgments. Bellman and Zadeh [19] had introduced the use of fuzzy set theory in MCDM and it proved to be an effective approach in dealing with uncertainty in human decision making process. Since then, it had become an important tool in constructing a decision making framework that incorporates subjective judgments that entails in the personnel selection process.

The main objective of this paper is to propose an approach to solve personnel selection process by using Hamming distance method. Inspired by algorithm proposed by Canós et al. [11], we extend and improve Canós's algorithm by adding weight in the classical Hamming distance. In our proposed method we suggest two types of weight which are subjective and objective weights. The linguistic terms correspondence to triangular fuzzy numbers are used to evaluate the performance rating values as well as the weight of the criteria, in which later will be expressed into interval valued fuzzy numbers. In this approach, we also identify the changes in ranking of the alternatives when different values of *α* are used. The remaining of this paper is organized as follows. The next section, we briefly explain the preliminary concerning fuzzy set and Hamming distance.  Section 3 will briefly explain about the Hamming distance method and subjective and objective weights. In Section 4, we propose a new algorithm for personnel selection problem. The new algorithm is called HDMSOW's.  Section 5 validates the HDMSOW's by conducting a numerical example. The last section concludes this paper.

## 2. Preliminaries

A fuzzy set *A* in *X* is defined as a set of ordered pairs (see [20]):

(1) $$\begin{array}{c} A=\left\{\langle x,\mu_{A}\left(x\right)\rangle :x\in X\right\}, \end{array}$$

where *X* is denoted as a universe of discourse and *μ*_*A*_(*x*) is the membership function of *A* defined as

(2) $$\begin{array}{c} \mu_{A}:X⟶\left[0,1\right]. \end{array}$$

A triangular fuzzy number is specified by three parameters and can be defined as triplet *A* = (*a*_1_, *a*_2_, *a*_3_), where *a*_1_ < *a*_2_ < *a*_3_ with the *x* = *a*_2_ as the core of the triangle. Its membership function can be represented as [21]

(3) $$\begin{array}{c} \mu_{A}\left(x\right)=\{\begin{array}{cc} 0, & x<a_{1},\\ \frac{\left(x-a_{1}\right)}{\left(a_{2}-a_{1}\right)}, & a_{1}\leq x\leq a_{2},\\ \frac{\left(a_{3}-x\right)}{\left(a_{3}-a_{2}\right)}, & a_{2}\leq x\leq a_{3},\\ 0, & x>a_{3}. \end{array} \end{array}$$

The *α*-cuts of this fuzzy number *A* are denoted by

(4) $$\begin{array}{c} {\left[A\right]}_{\alpha }=\left[a_{1}+\alpha \left(a_{2}-a_{1}\right),a_{3}-\alpha \left(a_{3}-a_{2}\right)\right],\;\alpha \in \left(0,1\right]. \end{array}$$

An interval-valued fuzzy set *A* in universe discourse *X* is denoted by (see [22, 23])

(5) $$\begin{array}{c} A=\left\{\left(x,\left[\mu_{A}^{L}\left(x\right),\mu_{A}^{U}\left(x\right)\right]\right)\;|\;x\in X\right\}, \end{array}$$

where *μ*_*A*_^*L*^(*x*), *μ*_*A*_^*U*^(*x*) : *X* → [0,1], *μ*_*A*_^*L*^(*x*) is lower bound, and *μ*_*A*_^*U*^(*x*) is upper bound of membership.

The multiplication of two interval-valued fuzzy numbers, *A* = [*a* ^*L*^, *a* ^*U*^] and *B* = [*b* ^*L*^, *b* ^*U*^], can be defined as (see [21])

(6) $$\begin{array}{c} A\cdot B=\left[a^{L},a^{U}\right]\cdot \left[b^{L},b^{U}\right]=\left[c^{L},c^{U}\right], \end{array}$$

where

(7) $$\begin{array}{c} c^{L}=\min\left\{a^{L}b^{L},a^{L}b^{U},a^{U}b^{L},a^{U}b^{U}\right\},\\ c^{U}=\max\left\{a^{L}b^{L},a^{L}b^{U},a^{U}b^{L},a^{U}b^{U}\right\}. \end{array}$$

Hamming distance methods to be used in this paper are presented as follows

**Definition 1 (see [24])**

Given two fuzzy subsets of *A* and *B* with a reference set, *X* = {*x*_1_, *x*_2_,…, *x*_*n*_} and memberships function *μ*_*A*_ and *μ*_*B*_.

Then the Hamming distance is defined as

(8) $$\begin{array}{c} d\left(A,B\right)=\overset{n}{\underset{j=1}{\sum }}\left|\mu_{A}\left(x_{j}\right)-\mu_{B}\left(x_{j}\right)\right|. \end{array}$$

The normalized Hamming distance for two interval-valued fuzzy numbers *A* and *B*, whose membership functions are as follows:

(9) μA(xj)=[axjL,axjU],  μB(xj)=[bxjL,bxjU],j=1,2,…,n.

is defined as

(10) $$\begin{array}{c} d_{\text{NHD}}\left(A,B\right)=\frac{1}{2n}\left(\overset{n}{\underset{j=1}{\sum }}\left(\left|a_{x_{j}}^{L}-b_{x_{j}}^{L}\right|+\left|a_{x_{j}}^{U}-b_{x_{j}}^{U}\right|\right)\right). \end{array}$$

**Definition 2 (see [25])**

The weighted Hamming distance of dimension *n* is a mapping *d*_WHD_ : [0,1]^*n*^ × [0,1]^*n*^ → [0,1] that associated with weighting vector *W* of dimension *n* with *W* = ∑_*j*=1_^*n*^, *w*_*j*_ = 1, and *w*_*j*_ ∈ [0,1]. Then the weighted Hamming distance is defined as

(11) $$\begin{array}{c} d_{\text{WHD}}\left(A,B\right)=\overset{n}{\underset{j=1}{\sum }}w_{j}\left|\mu_{A}\left(x_{j}\right)-\mu_{B}\left(x_{j}\right)\right|. \end{array}$$

According to [12] the weighted Hamming distance can be the normalized Hamming distance if *w*_*j*_ = 1/*n* for *j* = 1,2,…, *n*.

## 3. Hamming Distance Method and Subjective and Objective Weights

### 3.1. Hamming Distance Method

Hamming distance is one of the distance measures that can be applied in personnel selection process. This is due to its ability in calculating the distance between ideal alternative and alternative. The ideal alternative is a virtual alternative in which the criteria values are expressed as close as possible to ideal values which is rationale for human thinking to achieve. There are several methods that focus on identifying and measuring the ideal alternative. However this measurement is beyond our scope of research. In this paper, the evaluation on the ideal alternative is made based on assumption of the optimum value of each criterion that alternatives should achieve for the specified job. We also disregard the usage of maximum value, for example, (1,1, 1) in case of the triangular fuzzy number of all criteria evaluations. Rationally, it is hard for the alternatives to achieve a perfect score for some criteria especially when the evaluation of the criteria itself is made from human based judgment that mostly in subjective terms could be varied from one person to others. The ranking of alternatives is made through the comparison between the alternatives and the ideal alternative [26] such that, the alternatives with the minimum distance values are likely to be selected. However, when the distance values between the alternatives are the same, the decision makers will face a problem in ranking them. Thus, with the help of weights, it will help decision makers to distinguish between the criteria that valued the most for the specified job than the other criteria.

### 3.2. Subjective and Objective Weights

The decision makers are genuinely aware that they cannot assume that all criteria are equally important as it holds its own meaning and neediness, especially when its focus is only to one subject or position. For example, when recruiting the appropriate applicant for position credit officer, the criteria that might be valued most are experienced in credit analysis and personality assessment. Generally, the other criteria are also valuable but they are not as important as these two criteria. Plus it is a human nature to have diverse opinion in evaluating process. Thus it is undeniable that the criteria weight plays an important role in MCDM problem as it depicted the relative weightiness of the criteria must be assigned [3]. Alternatively, numerous approaches has been generalized and introduced to solve this problem. These methods can be categorized into two groups which are subjective and objective weights.

The subjective weight are determined solely based on the preference of the decision makers [27, 28]. These evaluations are basically based on experience, perception, and knowledge [29]. In a general view, it is a process of assigning subjective preferences to the criteria [29]. AHP method, eigenvector method, and weighted least square method can be used to calculate this approach. Beside, objective weight measured the weight with the use of mathematical models such as entropy method [30] and multiple objective programming [31]. This approach solves without any consideration from the decision makers preference. The use of objective weight can overcome some of the limitations in subjective weight such as inconsistency problem in subjective weight. Furthermore, it is useful when the reliable subjective weight is not available [29].

One of the objective weighting measure that vastly has been used in MCDM field is Shannon's entropy concept [32]. Shannon's entropy concept is a general measure of uncertainty in information formulated in terms of probability theory [30]. This concept is appropriate for calculating the relative contrast intensities of criteria to represent the average intrinsic information transmitted to the decision maker [33]. It began when Shannon first introduced the application of entropy in communication theory and since then, he had contributed the most fundamental definition of the entropy measure in the information theory [34]. This concept had been applied in wide range area exemplified mathematics [35], spectral analysis [36], and economics [37]. Entropy weight is a parameter that describes how much diverse alternatives approach one another with respect to a certain criteria [3, 28]. This concept is also, relatively known in the measurement of fuzziness [38]. Hence this method is suitable to be applied in our approach as we will deal with fuzzy data. Apart from that, the total weights for all criteria values will equal to one in which satisfy the condition that need in weighted Hamming distance method.

## 4. Hamming Distance Method with Subjective and Objective Weights

In this section, the description and algorithm for the HDMSOWs is constructed. To our best knowledge, the study of using a weighted Hamming distance method in solving personnel selection problem has rarely been done. Merigó and Gil-Lafuente [12] had presented a study involving the use of weighted Hamming distance method, integrated with Ordered Weighted Averaging (OWA) but without the use of fuzzy numbers. Hence, we would like to expand the use of weighted Hamming distance in personnel selection by using fuzzy data and we propose two types of weights which are subjective and objective weights. The elements of this HDMSOWs can be presented in the following descriptions.

Let us assume that there is a set of *m* possible alternatives, *A* = {*A*_1_, *A*_2_,…, *A*_*m*_} to be evaluated based on a set of *n* respective criteria, *C* = {*C*_1_, *C*_2_,…, *C*_*n*_}. These evaluations are done by a set of *m* decision makers, *E* = {*E*_1_, *E*_2_,…, *E*_*m*_} by using linguistic variables. To capture the linguistic terms, we use triangular fuzzy numbers. The linguistic variables are divided into two categories which are the evaluation on criteria weight and the evaluation on criteria. The given algorithm is unfolded as follows.

**Step 1 (construct a decision matrix for ideal alternative)**

The decision matrix for ideal alternative is given as follows:

(12) $$\begin{array}{c} I=\left[v_{1},v_{2},\ldots ,v_{n}\right]. \end{array}$$

The ideal alternative matrix represents the optimum values of *n* selection criteria *C* = {*C*_1_, *C*_2_,…, *C*_*n*_} that the alternatives should achieve. These values are set up by decision makers.

**Step 2 (construct a decision matrix for alternatives)**

The decision matrix for performance alternatives is given as follows:

(13) D=C1C2⋯CnA1A2⋮Am[x11x12⋯x1nx21x22⋯x2n⋮⋮⋯⋮xm1xm2⋯xmn]

where *x*_*ij*_ represent the linguistic assessment on the utility ratings of alternative *A*_*i*_  (*i* = 1,2,…, *m*) with respect to *n* selection criteria *C* = {*C*_1_, *C*_2_,…, *C*_*n*_} evaluated by the decision makers.

**Step 3 (construct a decision matrix for weight (criteria importance))**

The weighting matrix for criteria weight; *w*_*ij*_ evaluated by the decision makers, *E*_*i*_  (*i* = 1,2,…, *m*) is given as follows:

(14) W=E1E2⋮EmC1C2⋯Cn[w11w12⋯w1nw21w22⋯w2n⋮⋮⋯⋮wm1wm2⋯wmn]

The weighting matrix represents the relative importance of *n* selection criteria *C*_*j*_  (*j* = 1,2,…, *n*) given by the decision makers.

**Step 4 (construct an interval-valued fuzzy number)**

By using *α*-cut of triangular fuzzy number, the interval performance matrix for alternatives, ideal alternatives, and criteria weight are derived as follows, respectively

(i) The interval decision matrix for the ideal alternative:
  (15) $$\begin{array}{c} I_{\alpha }=\left[\left[{\left(v_{1}\right)}_{\alpha }^{L},{\left(v_{1}\right)}_{\alpha }^{U}\right],\left[{\left(v_{2}\right)}_{\alpha }^{L},{\left(v_{2}\right)}_{\alpha }^{U}\right],\ldots ,\left[{\left(v_{n}\right)}_{\alpha }^{L},{\left(v_{n}\right)}_{\alpha }^{U}\right]\right]. \end{array}$$
(ii) The interval decision matrix for performance alternatives:
  (16) $$\begin{array}{c} D_{\alpha }=\left[\begin{array}{cccc} \left[{\left(x_{11}\right)}_{\alpha }^{L},{\left(x_{11}\right)}_{\alpha }^{U}\right] & \left[{\left(x_{12}\right)}_{\alpha }^{L},{\left(x_{12}\right)}_{\alpha }^{U}\right] & \cdots & \left[{\left(x_{1n}\right)}_{\alpha }^{L},{\left(x_{1n}\right)}_{\alpha }^{U}\right]\\ \left[{\left(x_{21}\right)}_{\alpha }^{L},{\left(x_{21}\right)}_{\alpha }^{U}\right] & \left[{\left(x_{22}\right)}_{\alpha }^{L},{\left(x_{22}\right)}_{\alpha }^{U}\right] & \cdots & \left[{\left(x_{2n}\right)}_{\alpha }^{L},{\left(x_{2n}\right)}_{\alpha }^{U}\right]\\ \vdots & \vdots & \cdots & \vdots \\ \left[{\left(x_{m1}\right)}_{\alpha }^{L},{\left(x_{m1}\right)}_{\alpha }^{U}\right] & \left[{\left(x_{m2}\right)}_{\alpha }^{L},{\left(x_{m2}\right)}_{\alpha }^{U}\right] & \cdots & \left[{\left(x_{mn}\right)}_{\alpha }^{L},{\left(x_{mn}\right)}_{\alpha }^{U}\right] \end{array}\right]. \end{array}$$
(iii) The interval decision matrix for criteria weight
  (17) $$\begin{array}{c} W_{\alpha }=\left[\begin{array}{cccc} \left[{\left(w_{11}\right)}_{\alpha }^{L},{\left(w_{11}\right)}_{\alpha }^{U}\right] & \left[{\left(w_{12}\right)}_{\alpha }^{L},{\left(w_{12}\right)}_{\alpha }^{U}\right] & \cdots & \left[{\left(w_{1n}\right)}_{\alpha }^{L},{\left(w_{1n}\right)}_{\alpha }^{U}\right]\\ \left[{\left(w_{21}\right)}_{\alpha }^{L},{\left(w_{21}\right)}_{\alpha }^{U}\right] & \left[{\left(w_{22}\right)}_{\alpha }^{L},{\left(w_{22}\right)}_{\alpha }^{U}\right] & \cdots & \left[{\left(w_{2n}\right)}_{\alpha }^{L},{\left(w_{2n}\right)}_{\alpha }^{U}\right]\\ \vdots & \vdots & \cdots & \vdots \\ \left[{\left(w_{m1}\right)}_{\alpha }^{L},{\left(w_{m1}\right)}_{\alpha }^{U}\right] & \left[{\left(w_{m2}\right)}_{\alpha }^{L},{\left(w_{m2}\right)}_{\alpha }^{U}\right] & \cdots & \left[{\left(w_{mn}\right)}_{\alpha }^{L},{\left(w_{mn}\right)}_{\alpha }^{U}\right] \end{array}\right], \end{array}$$

where 0 ≤ *α* ≤ 1. The value of *α* represents the degree of confidences in the decision makers' assessment with respect to ideal alternative, alternatives rating, and criteria weights.

**Step 5 (calculating of criteria weight)**

The criteria weight of *n* selection criteria *C* = {*C*_1_, *C*_2_,…, *C*_*n*_} evaluated by the decision makers will be calculated using two methods, which are subjective and objective weights.

(a) Subjective weight. The subjective weight of *n* selection criteria *C* = {*C*_1_, *C*_2_,…, *C*_*n*_} may be considered as the average weights [9] and its calculation is [9, 28]
  (18) $$\begin{array}{c} w_{j}=\frac{1}{m}\left(\overset{m}{\underset{i=1}{\sum }}w_{ij}\right),\;i=1,2,...,m;\;j=1,2,\ldots ,n. \end{array}$$
(b) Objective weight. The interval valued fuzzy number is transformed into crisp number before using Shannon's entropy concept.

The crisp value of interval weight is given by [39]

(19) $$\begin{array}{c} w_{ij}=\frac{\left(w_{ij}^{l}+w_{ij}^{u}\right)}{2}. \end{array}$$

Then Shannon's entropy concept is used to obtain the weight.

The details of Shannon's entropy concept are defined as follows [27, 39].

*Step 5* *.1.* Normalized each criterion weight to obtain the projection value; *p*_*ij*_:

(20) $$\begin{array}{c} p_{ij}=\frac{w_{ij}}{\sum_{i=1}^{m}w_{ij}},\;i=1,\ldots ,m,\;\;j=1,\ldots ,n. \end{array}$$

Consequently, a projection matrix representing a relative weight of each criterion from the decision maker evaluation is expressed as

(21) $$\begin{array}{c} P=\left[\begin{array}{cccc} p_{11} & p_{12} & \cdots & p_{1n}\\ p_{21} & p_{22} & \cdots & p_{2n}\\ \vdots & \vdots & \cdots & \vdots \\ p_{m1} & p_{m2} & \cdots & p_{mn} \end{array}\right]. \end{array}$$

*Step 5.2.* Calculate entropy values *e*_*j*_ as

(22) $$\begin{array}{c} e_{j}=-k\overset{m}{\underset{i=1}{\sum }}p_{ij}\ln p_{ij},\;j=1,\ldots ,n, \end{array}$$

where *k* is constant and let *k* = (ln⁡*m*)^−1^. If *p*_*ij*_ = 0, then *p*_*ij*_ln⁡*p*_*ij*_ is equal to 0.

*Step 5.3*. Calculate the degree of diversification, *d*_*j*_:

(23) $$\begin{array}{c} d_{j}=1-e_{j},\;j=1,\ldots ,n. \end{array}$$

*Step 5.4*. Calculate the criteria weight, *w*_*j*_:

(24) $$\begin{array}{c} w_{j}=\frac{d_{j}}{\sum_{k=1}^{n}d_{k}}. \end{array}$$

**Step 6 (calculating the distance values)**

(a) Subjective weight. Before calculating the distance values, calculate the overall performance evaluation of ideal alternatives and alternatives by multiplying the aggregate weight with each criterion [21].

For the ideal alternative:

(25) $$\begin{array}{c} R_{j}=\left[v_{j}^{L},v_{j}^{U}\right]\cdot \left[w_{j}^{L},w_{j}^{U}\right]=\left[r_{j}^{L},r_{j}^{U}\right], \end{array}$$

and for the alternatives:

(26) Sij=[xijL,xijU]·[wjL,wjU]=[sijL,sijU],

where

(27) $$\begin{array}{c} r^{L}=\min\left\{v^{L}w^{L},v^{L}w^{U},v^{U}w^{L},v^{U}w^{U}\right\};\\ r^{U}=\max\left\{v^{L}w^{L},v^{L}w^{U},v^{U}w^{L},v^{U}w^{U}\right\};\\ s^{L}=\min\left\{x^{L}w^{L},x^{L}w^{U},x^{U}w^{L},x^{U}w^{U}\right\};\\ s^{U}=\max\left\{x^{L}w^{L},x^{L}w^{U},x^{U}w^{L},x^{U}w^{U}\right\}. \end{array}$$

Then, calculate the distance values between the ideal alternatives with the alternatives by using Definition 1:

(28) $$\begin{array}{c} d_{\text{NHD}}\left(R,S\right)=\frac{1}{2n}\left(\overset{n}{\underset{j=1}{\sum }}\left(\left|r_{j}^{L}-s_{ij}^{L}\right|+\left|r_{j}^{U}-s_{ij}^{U}\right|\right)\right). \end{array}$$

(b) Objective weight. For the objective weight, the distance values are calculated by using Definition 2:

(29) $$\begin{array}{c} d_{\text{WHD}}\left(I,D\right)=\overset{n}{\underset{j=1}{\sum }}\left(w_{j}\left|v_{j}^{L}-x_{ij}^{L}\right|+w_{j}\left|v_{j}^{U}-x_{ij}^{U}\right|\right). \end{array}$$

**Step 7 (ranking the candidate)**

The alternatives are ranked in ascending order according to the distance values for respective *α* values. The alternative with the less distance value is considered as the best choice.

**Step 8 (repeat Steps 4, 5, and 6 for different values of *α*)**

The alternatives are ranking according to the different values of *α*.

**Step 9 (selection of the appropriate alternative by the decision makers)**

* *

## 5. A Numerical Example

**Figure 1:**
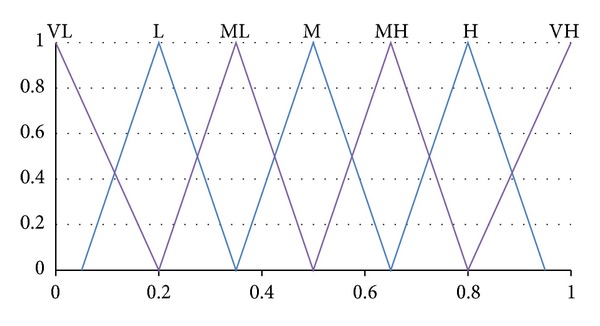
The fuzzy linguistic variables for each criterion weight.

**Figure 2:**
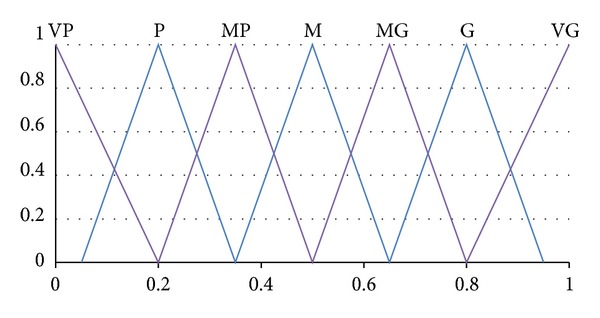
The fuzzy linguistic variables for each alternative.

An example on the personnel selection in an academic institution is provided to validate the proposed algorithm. Suppose that the academic institution intends to employ a lecturer based on consideration of four main criteria which are experienced in teaching areas (*C*_1_), proficiency in performing research (*C*_2_), personality assessment (*C*_3_), and past contribution (*C*_4_). Assume that after preliminary selection phase, four alternatives *A*_1_, *A*_2_, *A*_3_, and *A*_4_ are qualified for final evaluation. A committee of experts (decision makers) consisting of three persons is formed, namely, *D*_1_, *D*_2_, and *D*_3_. The information of this study is given in Figures 1 and 2 and Tables 1, 2, 3, 4, and 5, while the results from the numerical examples are shown in Tables 6, 7, 8, 9, and 10. As mentioned before, TOPSIS is one of the existing MCDM methods that can be used to solve personnel selection problem. Thus it can be used to validate the proposed method and the results by using this method that is shown in Table 11. More explanation on these figures and tables are explained in the discussion section.

### 5.1. Discussion

Based on the results obtained, the proposed HDMSOWs can be summarized as follows.

**Step 1**

Ideal alternative matrix (12) is built from the evaluations of the criteria based on linguistic variables taken from Wang and Lee [28] as illustrated in Figure 2 and Table 2. The linguistic terms are represented by triangular fuzzy number ranging from “very poor” to “very good.” Table 4 shows the decision makers evaluation on ideal alternative. In this paper, we assume that the *m* decision makers had come to an agreement in standardizing into one final value for each criterion.

**Step**

Decision matrix for alternatives evaluation on each criterion (13) is obtained by using the same linguistic variables adopted from Wang and Lee [28] as illustrated in Figure 2 and Table 2. Similar to Step 1, these terms are captured in the form of the triangular fuzzy number. The alternatives performance evaluations are ranging from “very poor” to “very good.” Table 5 illustrates each fuzzy linguistic term to its corresponding fuzzy number for each alternative.

**Step 3**

The weighting matrix (14) for each criterion is evaluated and determined by the decision makers based on linguistic variables pictured in Figure 1 and Table 1. Like the previous step, these linguistic terms are expressed in the form of triangular fuzzy numbers and are ranging from “very low” to “very high.” Table 3 marks the evaluation of the criteria weights by the decision makers according to their own judgment in evaluating criteria's importance for the specified job.

**Step 4**

By using the *α*-cuts of fuzzy numbers, the interval value of the fuzzy number of the performance matrics for the ideal alternative (15), the alternatives (16), and criteria weight (17) are built. The values of *α* show the degree of confidences for the decision makers in evaluating the criteria performance of each alternative.

**Step 5**

The objective and subjective weights are identified. The subjective weight is measured based on (18).  Table 6 shows the subjective weight for each criterion at *α* = 0 and *α* = 0.5. While for objective weight, Shannon's entropy concept (19)–(24) is used to obtain the weight. The projection values are shown in Table 7.  Table 8 consist of entropy values (*e*_*j*_), degree of diversifications (*d*_*j*_), and the objective weight (*w*_*j*_). The use of objective weight will give an insight to the decision maker in determining which criteria is needed the most in which *C*_3_  and *C*_4_ are considered as the most important criteria based on Shannon's entropy concept. It is known that objective weight can be obtained without consideration of decision maker's preferences; however, since the evaluation of criteria weight exists, the objective weight is obtained based on the evaluation of criteria weight.

**Step 6**

The distance values between the ideal alternative and the alternatives are calculated by using the Hamming distance method. For the subjective weight, the overall performance evaluation for the ideal alternative (25) and the alternatives (26) are determined beforehand the use of the normalized Hamming distance method (28). For the objective weight, the distance values are obtained from the use of the weighted Hamming distance method (29). The distance values show how much is the similarity between the alternatives and the ideal alternative.

**Step 7**

The ranking of the alternatives is made based on the distance values obtained before. The alternative with the less distance value is considered as a preferable alternative to be selected.  Table 9 shows the distance value for each alternative at *α* = 0 and *α* = 0.5.  Table 10 shows the ranking of the alternatives based on the distance values with the use of subjective and objective weights.

**Step 8**

The steps are repeated by using different values of *α*, *α* ∈ [0,1]. Under different values of *α*, the decision makers may expect the different outcome in the ranking of the alternatives. If there exist two or more alternatives on the same ranking which indicate that they having the same distance values, the decision makers may refer to the criteria weight, which mean the alternative that perform well in the criteria that is needed the most is likely to be selected.

**Step 9**

The decision makers then will select the suitable alternative to fill the vacancy based on the ranking of the alternatives. The decision makers also can make the decision based on the preferable *α*  levels since the ranking may be changed at the different values of *α*. Apparently, the most suitable alternative for the post by using both subjective and objective weights is the alternative with the minimum distance values. From Table 10, *A*_4_ is likely to be selected by the decision makers regarding his/her distance values. Here, we also present the results by using TOPSIS method to validate the proposed approach. Consequently, the same results are recorded by using TOPSIS method in which *A*_4_ is the possible alternative to be selected. The ranking for the other alternatives also can be clarified as almost similar to the results by using the proposed method.

## 6. Conclusions

In this paper, we have presented a novel approach of handling personnel selection process by using the Hamming distance method. Based on the fact that most of criteria assessment is in qualitative or in subjective measurement, fuzzy set theory has been applied to overcome this limitation. Furthermore, realizing the importance of weighting the criteria in determining which criteria are valued the most; two types of weights have been applied in this paper which are objective and subjective weights. The objective weight is determined by the application of Shannon's entropy concept and the subjective weight is obtained based on the preference of the decision maker. With the use of the weighted Hamming distance, the distance values between the ideal alternative and the alternatives are identified and the ranking of the alternatives based on the overall evaluation of the criteria is made. The final results showed that the criteria *C*_3_ and *C*_4_ are considered as the important criteria and  *A*_4_  is considered as the best alternative to choose based on the use of subjective and objective weights. With emphasis on finding the distance measure between ideal alternative and alternatives with the use of subjective and objective weights, our method provides an effective way to be used. In addition, we are also incorporating fuzzy linguistic terms to express the subjective assessment that the decision makers often exhibit, while evaluating the alternatives performance in certain criteria. We also provided the numerical example to prove the validity of this approach. To verify the proposed method, the TOPSIS method is used to compare the result and we can justify that the final results are almost the same for both methods. The proposed method also can overcome some limitation in the existing methods of MCDM that are involved with the inconsistency of judgement when there are the addition of alternatives and criteria. For further research, we are going to study the appropriate methods in evaluating ideal alternatives hence improving the HDMSOWs.

## Figures and Tables

**Table 1:** Fuzzy linguistic terms and respective fuzzy numbers for each criterion weight.

Linguistic terms	Fuzzy numbers
Very low (VL)	(0, 0, 0.2)
Low (L)	(0.05, 0.2, 0.35)
Medium low (ML)	(0.2, 0.35, 0.5)
Medium (M)	(0.35, 0.5, 0.65)
Medium high (MH)	(0.5, 0.65, 0.8)
High (H)	(0.65, 0.8, 0.95)
Very high (VH)	(0.8, 1, 1)

**Table 2:** Fuzzy linguistic terms and respective fuzzy numbers for each criterion.

Linguistic terms	Fuzzy numbers
Very poor (VP)	(0, 0, 0.2)
Poor (P)	(0.05, 0.2, 0.35)
Medium poor (MP)	(0.2, 0.35, 0.5)
Fair (F)	(0.35, 0.5, 0.65)
Medium good (MG)	(0.5, 0.65, 0.8)
Good (G)	(0.65, 0.8, 0.95)
Very good (VG)	(0.8, 1, 1)

**Table 3:** Decision makers' evaluation on each criterion weight.

Criteria	*C*_1_	*C*_2_	*C*_3_	*C*_4_
*D*_1_	VH	H	H	MH
*D*_2_	H	VH	MH	H
*D*_3_	VH	VH	H	H

**Table 4:** Decision makers' evaluation on ideal alternative.

Criteria	*C*_1_	*C*_2_	*C*_3_	*C*_4_
*I*	VG	G	VG	MG

**Table 5:** Decision makers rating on alternative performance.

Alternatives	*C*_1_			*C*_2_			*C*_3_			*C*_4_		
	*D*_1_	*D*_2_	*D*_3_	*D*_1_	*D*_2_	*D*_3_	*D*_1_	*D*_2_	*D*_3_	*D*_1_	*D*_2_	*D*_3_
*A*_1_	G	G	F	F	MG	F	G	VG	VG	G	VG	MG
*A*_2_	F	G	G	F	F	F	G	MG	G	MG	G	G
*A*_3_	F	VG	F	MG	VP	G	VG	G	MG	VG	G	G
*A*_4_	G	G	G	MG	G	G	VG	VG	VG	G	G	MG

**Table 6:** Subjective weight for each criterion at *α* = 0 and *α* = 0.5.

Criteria	*α* = 0	*α* = 0.5
*C*_1_	(0.75, 0.9833)	(0.84166, 0.95833)
*C*_2_	(0.75, 0.9833)	(0.84166, 0.95833)
*C*_3_	(0.60, 0.90)	(0.675, 0.825)
*C*_4_	(0.60, 0.90)	(0.675, 0.825)

**Table 7:** Each criterion projection value at *α* = 0 and *α* = 0.5.

Criteria	*D*_1_		*D*_2_		*D*_3_	
	*α* = 0	*α* = 0.5	*α* = 0	*α* = 0.5	*α* = 0	*α* = 0.5
*C*_1_	(0.34615)	(0.35185)	(0.30769)	(0.29630)	(0.34615)	(0.35185)
*C*_2_	(0.30769)	(0.29630)	(0.34615)	(0.35185)	(0.34615)	(0.35185)
*C*_3_	(0.35556)	(0.35556)	(0.28889)	(0.28889)	(0.35556)	(0.35556)
*C*_4_	(0.28889)	(0.28889)	(0.35556)	(0.35556)	(0.35556)	(0.35556)

**Table 8:** Shannon's entropy based weight.

Criteria	*e*_*j*_		*d*_*j*_		*w*_*j*_	
	*α* = 0	*α* = 0.5	*α* = 0	*α* = 0.5	*α* = 0	*α* = 0.5
*C*_1_	0.99864	0.99713	0.00136	0.00287	0.12385	0.20439
*C*_2_	0.99864	0.99713	0.00136	0.00287	0.12385	0.20439
*C*_3_	0.99585	0.99585	0.00415	0.00415	0.37615	0.29561
*C*_4_	0.99585	0.99585	0.00415	0.00415	0.37615	0.29561

**Table 9:** Distance value of subjective and objective weights at *α* = 0 and *α* = 0.5.

Distance	*A*_1_		*A*_2_		*A*_3_		*A*_4_	
	*α* = 0	*α* = 0.5	*α* = 0	*α* = 0.5	*α* = 0	*α* = 0.5	*α* = 0	*α* = 0.5
Subjective	0.12604	0.13993	0.15181	0.17915	0.17305	0.20001	0.04979	0.06338
Objective	0.23685	0.32263	0.31193	0.40220	0.36193	0.45220	0.11239	0.14087

**Table 10:** Ranking of alternatives at *α* = 0 and *α* = 0.5 by using HDMSOW's.

Ranking	Subjective weight		Objective weight	
	*α* = 0	*α* = 0.5	*α* = 0	*α* = 0.5
1	*A*_4_	*A*_4_	*A*_4_	*A*_4_
2	*A*_1_	*A*_1_	*A*_1_	*A*_1_
3	*A*_2_	*A*_2_	*A*_2_	*A*_2_
4	*A*_3_	*A*_3_	*A*_3_	*A*_3_

**Table 11:** Ranking of alternatives at *α* = 0 and *α* = 0.5 by using TOPSIS.

Ranking	Subjective weight		Objective weight	
	*α* = 0	*α* = 0.5	*α* = 0	*α* = 0.5
1	*A*_4_	*A*_4_	*A*_4_	*A*_4_
2	*A*_1_	*A*_1_	*A*_1_	*A*_1_
3	*A*_3_	*A*_3_	*A*_3_	*A*_3_
4	*A*_2_	*A*_2_	*A*_2_	*A*_2_
